# High left ventricular mass associated with increased risk of incident diabetes

**DOI:** 10.1038/s41598-023-50845-3

**Published:** 2024-01-02

**Authors:** Shih-Ming Chuang, Sung-Chen Liu, Ching-Hsiang Leung, Yuan-Teh Lee, Kuo-Liong Chien

**Affiliations:** 1 Institute of Epidemiology and Preventive Medicine, College of Public Health, National Taiwan University, Taipei, Taiwan; 2 Division of Endocrinology and Metabolism, Department of Internal Medicine, Mackay Memorial Hospital, Taipei, Taiwan; 3 Department of Medicine, Mackay Medical College, Taipei, Taiwan; 4 Mackay Junior College of Medicine, Nursing, and Management, Taipei, Taiwan; 5 Department of Internal Medicine, National Taiwan University Hospital, Taipei, Taiwan

**Keywords:** Endocrinology, Risk factors

## Abstract

Evidence for the role of electrocardiography or echocardiography in determining left ventricular hypertrophy for the risk of diabetes is still controversial. We aimed to explore whether left ventricular mass, as measured by these methods, is associated with the risk of diabetes in a community population. We recruited 2696 participants aged 35 years or older without diabetes who had undergone screening with electrocardiography and echocardiography. Left ventricular mass index (LVMI) was calculated using a formula, and participants were divided into tertiles based on their LVMI tertiles. During a median follow-up period of median, 8.9 years, a total of 405 participants developed diabetes. The incidence and risk of diabetes significantly increased with higher LVMI tertiles. Multivariate Cox regression analysis demonstrated that individuals in the highest LVMI tertile had a greater likelihood of developing incident diabetes, with a hazard ratio of 1.40 (95% CI 1.06–1.91), even after adjusting related covariates. The highest risk of diabetes was observed in the presence of both the uppermost LVMI tertile and electrocardiographically determined left ventricular hypertrophy for the Chinese population. Left ventricular hypertrophy identified by either electrocardiography or echo may serve as a surrogate marker for identifying the risk of diabetes in clinical practice.

## Introduction

Diabetes is associated with an increased risk of cardiovascular disease and all-cause mortality^1^. The prevalence of diabetes is growing rapidly due to unhealthy lifestyles and lack of exercise. Primary prevention for incident type 2 diabetes is essential through the early identification of related risk factors^2^. Well-recognized diabetes risk factors, such as obesity and insulin resistance not only increase the risk of developing diabetes but are also risk factors for left ventricular hypertrophy (LVH)^3^. The association between type 2 diabetes and subsequent LVH has been extensively studied^4^, with previous research showing that the presentation of LVH is common in individuals with type 2 diabetes and is highly associated with adverse cardiovascular events such as coronary disease, stroke^5^, and sudden death^6^ and the occurrence of congestive heart failure^7^. LVH has been occasionally observed even in early diabetes or pre-diabetes and is often correlated with insulin resistance in obese or hypertensive individuals^8,9^. Increased left ventricular mass could be associated with type 2 diabetes^10^. Clinically, LVH can be diagnosed by two entities, including electrocardiography (ECG) and echocardiography (Echo). Although they can often overlap and may be clinically distinct entities, each can provide different prognostic and underlying mechanistic information^11^. A 25-year cohort study demonstrated that higher electrocardiographically determined left ventricular hypertrophy(ECG-LVH) was positively associated with the risk of incident diabetes in a young, non-obese population^12^. Other research have noted that the improvements in ECG-LVH induced by hypertension therapy were associated with reduced diabetes incidence^13^. Although there have been several studies investigating the relationship between ECG-LVH and increased risk of diabetes, evidence regarding whether LVH is a risk factor for diabetes remains inconclusive. In order to investigate this hypothesis, we employed a combination of ECG and Echo to examine whether higher left ventricular mass is associated with an increased incidence of diabetes.

## Methods

### Study population

Of the 3602 participants from the Chin-Shan Community Cardiovascular Cohort (CCCC) study who participated in the baseline survey, 47.3% were men and 52.7% were women and about 36% of the population were more than 35 years in 1990, at baseline. Since the trial began in 1990, all recruited participants were personally surveyed through validity questionnaires and individual interviews. Participants was invited to join this study and physical examination and laboratory study were conducted by the physician. We collected and evaluated related information about lifestyle and anthropometric measurements every 2 years regularly during first 6 years of the trial and all the data were recorded and categorized in detail. Participants blood were collected to measure serum biochemical data, including glucose, lipid profile, liver function, and renal function, and repeat measurements were performed during subsequent follow-up until 2010.

Diabetes screening was utilized by checking fasting blood glucose. Besides, a 75-g oral glucose tolerance test was performed and glucose and insulin levels were collected 2 h after the glucose load.

Participants were excluded if they met any of the following criteria: incomplete blood data, pre-existing coronary artery disease, baseline stroke, type 2 diabetes or valvular heart disease. Due to the increased risk of left ventricular hypertrophy in patients with coronary artery disease, baseline stroke, or valvular heart disease, we were concerned about the potential overestimation of the patient's exposure risk; therefore, we excluded individuals with these factors.

**Figure 1:**
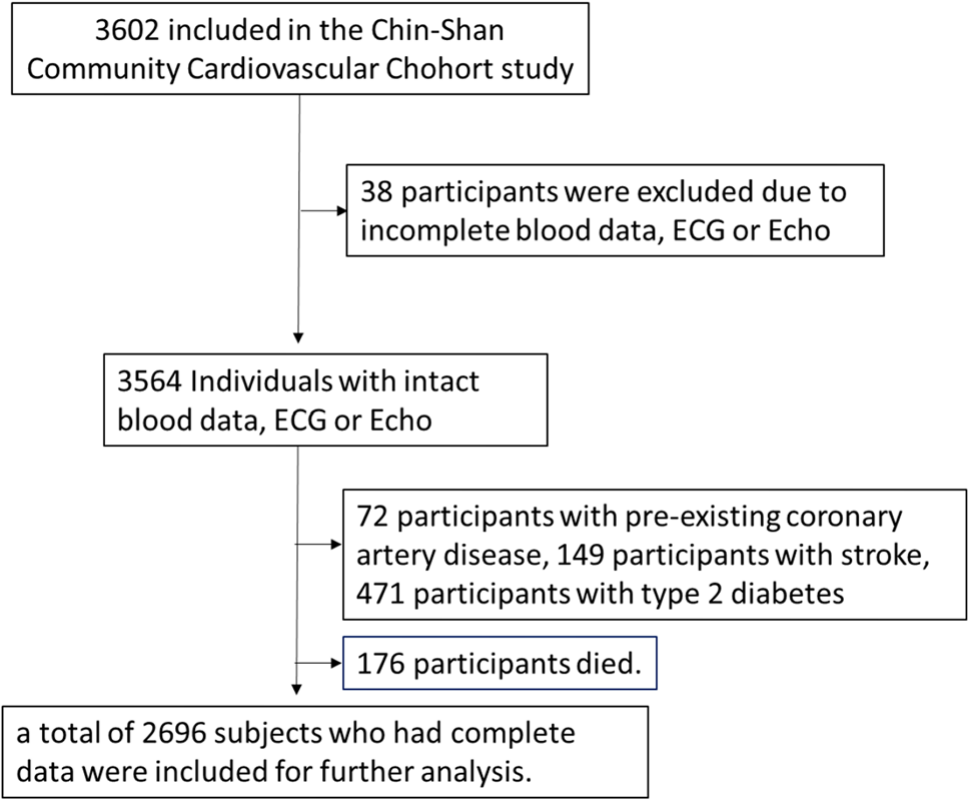
The flow diagram of participants.

Of the 3602 participants from the CCCC cohort, 38 participants were first excluded due to incomplete blood data, EKG or Echo. Subsequently, we excluded 72 participants with pre-existing coronary artery disease, 149 participants with a history of stroke, 473 participants with type 2 diabetes and 176 participants who died. Finally, a total of 2696 subjects who had complete data were included for further analysis (Fig. 1). We carried out all methods in accordance with relevant guidelines and regulations. In addition, we obtained informed consent from the subjects. The Research Ethics Committee of the National Taiwan University Hospital approved this study (Number: 202108099RINA).

### Left ventricular mass index (LVMI)

LVMI was utilized for the analysis of left ventricular mass. LVMI was derived from the modified American Society of Echocardiography-cube method^14^. Standard M-mode echocardiographic measurement were performed for the enrolled participants by qualified cardiologists during the 1992–1993 follow-up period. LVMI was calculated by dividing the left ventricular mass by the body surface area. We classified participants on the basis of tertiles of LVMI as tertile1 (< 100.9 g/m^2^), tertile2 (100.9–128.7 g/m^2^) and tertile3 (> 128.7 g/m^2^), respectively.

### ECG-LVH

ECG-LVH was identified by ECG. Electrocardiogram were recorded at baseline and simultaneous 12-lead ECGs were obtained from each recruited subject after resting for 10 s. ECG-LVH was defined by Cornell voltage criteria or by Sokolow-Lyon voltage criteria^15^.

### Confounding factors

Hypertension in subjects was diagnosed as systolic blood pressure > 140 mmHg and/or diastolic blood pressure > 90 mmHg, or receiving antihypertension medications. The diagnostic criteria for hyperlipidemia were defined by the National Cholesterol Education Program Adult Treatment Panel III or based on the use of lipid-lowering agents^16^. The HOMA-IR (Homeostasis Model Assessment of Insulin Resistance) is calculated using the formula [(Fasting plasma glucose in mmol/L) multiplied by (Fasting insulin in mU/L)] divided by 22.5^17^. The result obtained from this calculation provides an estimate of insulin resistance, with elevated HOMA-IR values suggesting an increase in insulin resistance.

### Outcome variables

We defined diabetes as fasting plasma glucose ≥ 126 mg/dL (7.0 mmol/L) or receiving anti-diabetic medication. We also defined the follow-up period as the period from baseline (1990) until one of following: new-onset diabetes, end of follow-up (2010), death or loss follow-up.

### Statistical analysis

The frequency and percentage of baseline characteristics was presented as categorical variables and mean SD was presented as a continuous variable. The Wilcoxon rank-sum test or ANOVA was applied to confirm statistical significance for continuous variables and Chi-square method for categorical variables. We calculated the incidence rates of diabetes and compared the association between the risk of incident diabetes and ECG-LVH or LVMI tertile, respectively. Cumulative incidence of diabetes was estimated by Kaplan–Meier curve according to either ECG-LVH or/and LVMI tertile, and Log-rank test was performed for the comparison of the difference in estimates. Kappa statistics were performed to analyze agreement among cross-classified categories by ECG and Echo finding^17^. Cox proportional hazards regression analyses were used to estimate the adjusted hazard ratios^18^ for incidence of diabetes. Univariable Cox regression was used to examine the association between ECG-LVH or LVMI tertiles or both, and the risk of incident diabetes. We used multivariable models to adjust for potential confounding variables; model 1 was adjusted for age and gender; model 2 was adjusted model 1 covariates plus hypertension, dyslipidemia and body mass index, and model 3 was adjusted for model 2 covariates plus HOMA-IR and glucose. Restricted cubic splines with the corresponding confidence limits were used to evaluate the effect of the continuous variable of interest (LVMI) on the risk of incident diabetes. Statistical analysis was performed using IBM SPSS release V.21.0 (IBM, Armonk, New York, USA).

## Results

During a median follow-up period of 8.9 years, 405 out of the total participants developed diabetes. The distribution of participants across tertiles was as follows: tertile1 (34.4%), tertile2 (33.4%), and tertile3 (32.1%), respectively. ECG-LVH was detected in 293 (10.8%) individuals. Baseline characteristics stratified by LVMI tertiles and ECG-LVH are shown in Table 1. Participants with tertile3 or ECG-LVH were found to be older, more likely to smoke, and had a higher proportion of males compared to those without ECG-LVH or in the other tertiles. Additionally, they had higher blood pressure, glucose, and creatinine levels. Moreover, participants in the higher tertile also had higher BMI, lipid profiles, and HOMA-IR.

**Table 1:** Clinical characteristics and laboratory data of individuals according to ECG-LVH or LVMI tertiles.

	Tertile1^a^ (n = 928)	Tertile2^a^ (n = 902)	Tertile3^a^ (n = 866)	p*	No ECG-LVH (n = 2403)	ECG-LVH (n = 293)	p*
Age (years)	49.1 ± 10.3	51.6 ± 10.4	55.0 ± 10.6	< 0.001	53.8 ± 12.3	59.4 ± 11.3	< 0.001
Gender (male, %)	23.4	43.9	61.6	< 0.001	43.9	60.6	< 0.001
Smoking (%)	15.2	29.8	38.0	< 0.001	39	51	< 0.001
BMI (kg/m^2^)	22.5 ± 3.0	23.6 ± 3.0	25.0 ± 3.3	< 0.001	23.4 ± 3.4	23.5 ± 3.4	0.580
SBP (mmHg)	116.8 ± 16.1	122.7 ± 17.2	129.5 ± 20.8	< 0.001	123.0 ± 18.9	136.0 ± 23.8	< 0.001
DBP (mmHg)	73.6 ± 9.5	77.0 ± 10.5	79.8 ± 11.6	< 0.001	76.2 ± 10.7	81.0 ± 12.1	< 0.001
Glucose(mg/dL)	104.5 ± 21.3	108.0 ± 26.3	111.8 ± 29.4	< 0.001	109.6 ± 9.7	112.5 ± 9.8	0.028
TC (mg/dL)	195.5 ± 43.2	199.8 ± 44.7	202.4 ± 47.0	0.015	197.2 ± 45.2	201.2 ± 46.8	0.484
TG (mg/dL)	107.6 ± 76.0	126.8 ± 92.0	143.0 ± 110.6	< 0.001	125.0 ± 68.4	130 ± 78.1	0.292
LDL-C (mg/dL)	133.8 ± 41.6	140.3 ± 43.2	143.6 ± 45.9	< 0.001	137.5 ± 95.0	133.8 ± 93.9	0.249
HDL-C (mg/dL)	49.9 ± 12.4	47.3 ± 11.6	45.2 ± 12.4	< 0.001	47.5 ± 12.7	47.5 ± 12.8	0.99
Cr (mg/dL)	0.78 ± 0.2	0.83 ± 0.2	0.87 ± 0.3	< 0.001	0.8 ± 0.3	0.9 ± 0.5	0.04
GPT (mg/dL)	45.4 ± 30.6	46.0 ± 20.6	49.5 ± 25.1	0.006	46.8 ± 25.1	47.2 ± 24.9	0.761
HOMA-IR	1.7 ± 2.1	1.9 ± 2.4	2.4 ± 5.9	0.010	2.0 ± 2.1	2.3 ± 6.6	0.061
LVMI (g/m^2^)	86.5 ± 14.3	117.2 ± 13.2	158.6 ± 29.3	< 0.001	115.0 ± 33.0	139.1 ± 39.3	0.001

Data are presented as mean value ± standard deviation or %.

*BMI* body mass index, *FPG* fasting plasma glucose, *PPG* post-prandial plasma glucose, *HbA1c* glycosylated hemoglobin, *TC* total cholesterol, *TG* triglyceride, *LDL-C* low-density lipoprotein cholesterol, *HDL-C* high-density lipoprotein cholesterol, *SBP* systolic blood pressure, *DBP* diastolic blood pressure, *GPT* glutamic-pyruvic transaminase, *Cr* creatinine, *HOMA-IR* Homeostasis Model Assessment of Insulin Resistance, *LVMI* left ventricular mass index.

*Statistical significance for continuous variables was confirmed by ANOVA or Wilcoxon rank-sum test, and categorical variables were tested using Chi-square method.

^a^Ranges of tertiles of LVMI: tertile1: < 100.9 g/m^2^, Tertile2: 100.9–128.7 g/m^2^, tertile3: > 128.7 g/m^2^.

The incidence of diabetes increased significantly across the tertiles of LVMI, as shown in Table 2. Cox regression analysis was performed, and after adjusting for age and sex, the HR for developing diabetes were 1.35 and 1.78 for LVMI tertiles2 and 3, respectively. Notably, the high LVMI group in tertile3 had a 40% higher risk of developing incident diabetes, with an HR of 1.40 (95% CI 1.06–1.91), even after adjusting for age, sex, hypertension, dyslipidemia, BMI, HOMA-IR and glucose. Furthermore, the presence of ECG-LVH was found to be associated with an increased risk of incident diabetes with a HR of 1.35 (95% CI 1.05–1.74), independent of age, sex, hypertension, dyslipidemia, BMI, and HOMA-IR, as detailed in Table 3.

**Table 2:** Number of study participants, incident cases, person-years, rates, and adjusted HRs (95% CI) by tertiles of echocardiographically determined left ventricular mass.

	Tertile1	Tertile2	Tertile3
Participants, n	928	902	866
Incident case, n	111	142	152
Person-years, n	5589.9	5127.0	4231.1
Incidence Rate/1000	19.8	27.7	35.9
HR, Model 1^a^	1 [Reference]	1.35 (1.02–1.78)	1.78 (1.33–2.38)
HR, Model 2^b^	1 [Reference]	1.22 (0.92–1.61)	1.44 (1.06–1.95)
HR, Model 3^c^	1 [Reference]	1.13 (0.85–1.50)	1.40 (1.06–1.91)

Multi-variate Cox regression model.

Model 1: age and sex.

Model 2: Model 1 + hypertension, dyslipidemia, BMI.

Model 3: Model 2 + HOMA-IR + glucose.

**Table 3:** Number of study participants, incident cases, person-years, rates, and HR by clinical characteristics and laboratory data of individuals with and without left ventricular hypertrophy.

	No ECG-LVH	ECG-LVH
Participants, n	2403	293
Incident case, n	335	70
Person-years, n	17,686.9	1961.9
Incidence rate/1000	25.0	37.7
HR, Model 1^a^	1 [Reference]	1.70 (1.26–2.28)
HR, Model 2^b^	1 [Reference]	1.62 (1.20–2.19)
HR, Model 3^c^	1 [Reference]	1.35 (1.05–1.74)

Multi-variate Cox regression model.

Model 1: age and sex.

Model 2: Model 1 + hypertension, dyslipidemia, BMI.

Model 3: Model 2 + HOMA-IR + glucose.

**Figure 2:**
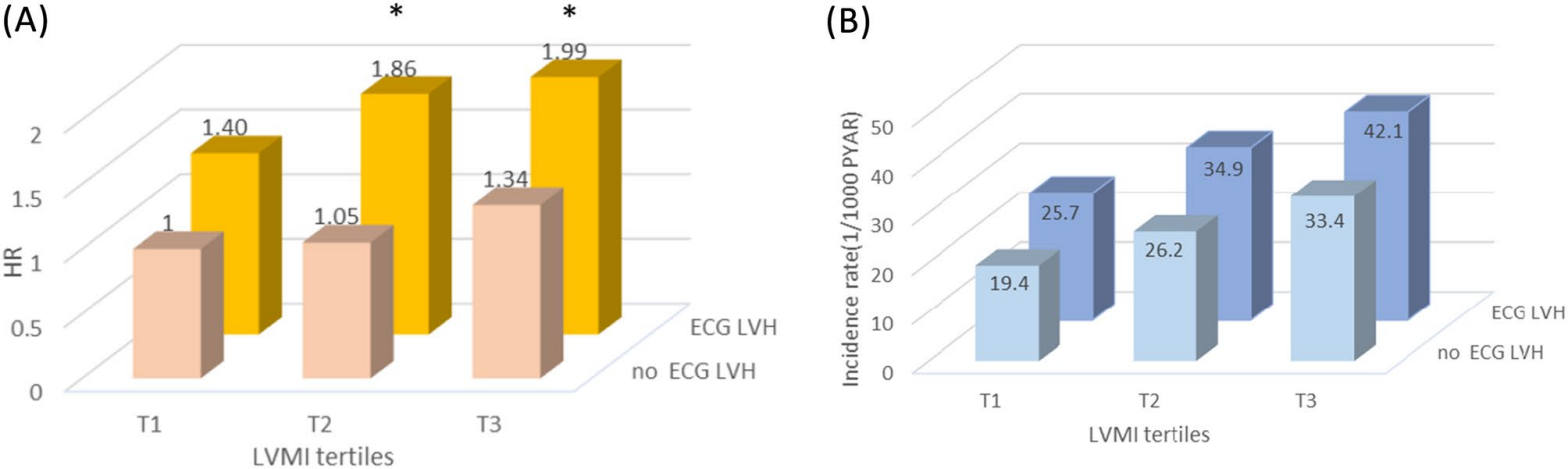
(**A**) HRs of incident diabetes adjusted for age, sex, hypertension, dyslipidemia, BMI, glucose and HOMA-IR and (**B**) incidence rate of diabetes in groups based on Echo-LV mass tertile and the presence or absence of ECG-LVH during 20 years of follow-up. *p < 0.05 compared with reference group.

**Figure 3:**
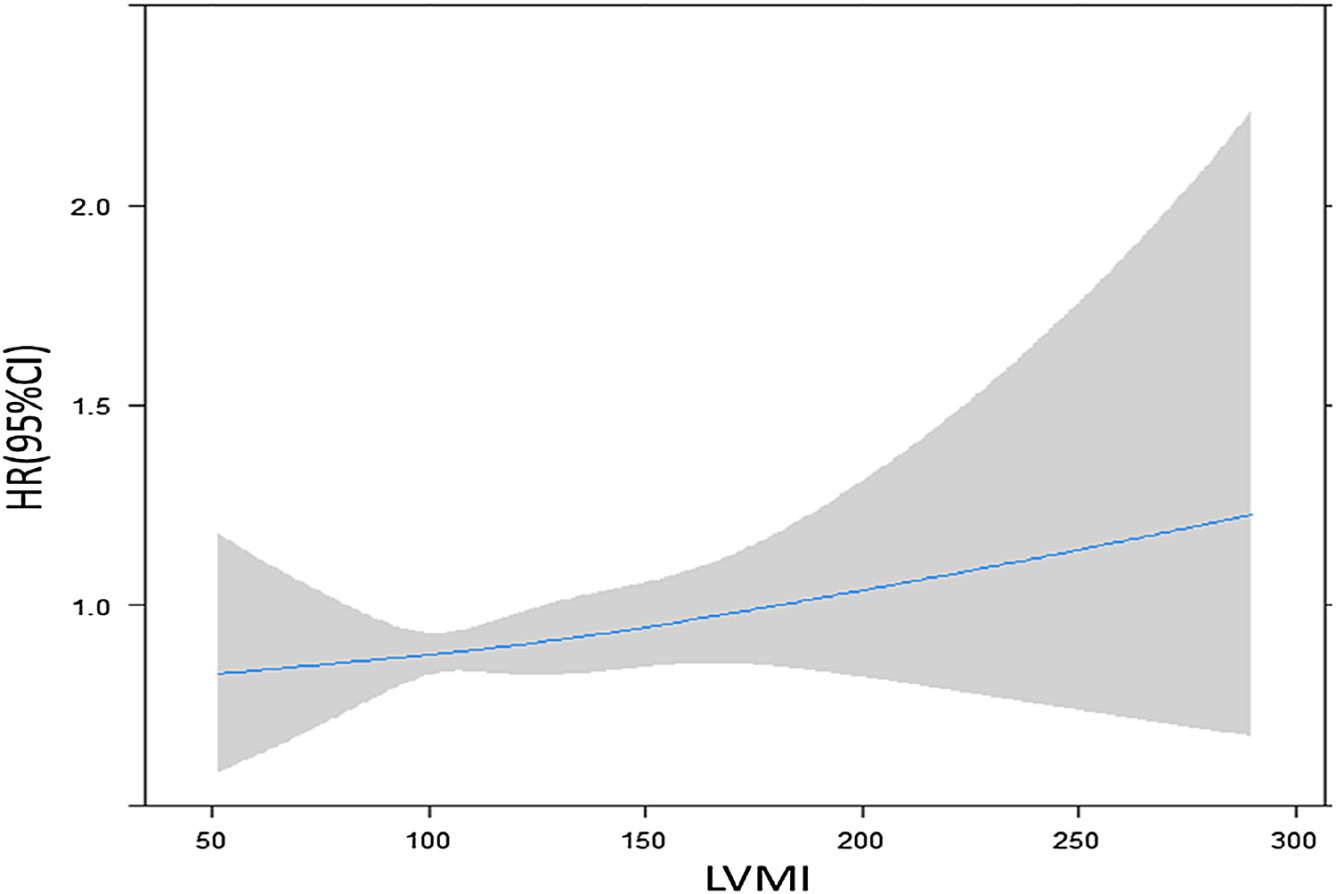
The association between LVMI and the risk of incident diabetes, analyzed using a spline regression model and adjusted for age, sex, hypertension, dyslipidemia, BMI, HOMA-IR, and glucose. The curves depict diabetes risk in relation to LVMI (50–300 g/m^2.7^), representing the linear component of proportional hazards models adjusted for key factors.

To gain a more comprehensive understanding of the relationship between these variables and the risk of diabetes, we conducted joint analyses by dividing patients into six groups based on ECG and Echo findings. The kappa value of 0.01 for ECG-LVH and LVMI tertile indicated poor concordance between the two tests. The reference group consisted of patients with tertile1 of LVMI and no ECG-LVH. Cox regression analysis was performed to assess the association of the cross-classified categories of ECG-LVH and LVMI tertiles with the risk of incident diabetes, and the results are presented in Fig. 2. These analyses complement each other and provide a comprehensive description of the synergistic interaction between these two variables and the risk of diabetes. The HR and incidence rate for the risk of diabetes are shown in Fig. 2 for all outcomes. Within either the ECG-LVH or no ECG-LVH category, a higher LVMI tertile was associated with a higher risk of diabetes. Similarly, within each tertile stage, ECG-LVH was associated with a higher risk of diabetes compared to no ECG-LVH. Spline regression analysis of the association between LVMI and the risk of diabetes revealed that the risk of diabetes increased gradually with increasing LVMI (Fig. 3).

## Discussion

Our study is the first to explore a positive association between LVMI tertiles and the risk of incident type 2 diabetes in the Chinese population. Compared with studies that solely used ECG, our study combined ECG and echo to investigate the association between left ventricular mass and the risk of diabetes. Our results showed that ECG-LVH or high LVMI was associated with an increased risk of diabetes compared to those without LVH or low LVMI. After adjusting for relevant covariates, increased LVMI was significantly associated with a higher risk of incident diabetes. Moreover, the coexistence of tertile3 and ECG-LVH had the highest risk of incident diabetes.

Some cross-sectional studies have suggested that the change in left ventricular mass is related to blood glucose control, and high HbA1c levels may lead to an increase in left ventricular mass^19^. In one study, multivariate regression analysis showed that type 2 diabetes was associated with LVMI and positively correlated with fasting blood glucose and HbA1c levels^10^. The Strong Heart Study found that early cardiac damage in T2DM was characterized by an increase in left ventricular mass, which may be a preclinical marker of myocardial structural changes and subclinical left ventricular dysfunction^20^. Additionally, high blood glucose may have a negative impact on left ventricular mass, even before the onset of overt type 2 diabetes. Pre-diabetes conditions such as insulin resistance, impaired fasting glucose, and impaired glucose tolerance have been found to be associated with increased left ventricular mass^21^.

Cohort observed studies have disclosed an association between LVH and incident diabetes among several populations, such as hypertension, obesity and even the young without any comorbidity. In a more than 1 year follow-up study of 4176 hypertensive non-diabetes age 58.7 years, the presence of LVH was associated with higher risk of diabetes. Cox regression showed LVH slightly increased HR 1.03 [95% CI, 1.01–1.005, *p* < 0.01] for incident diabetes compared of subjects without LVH^22^. In another 4-year follow-up study of 2887 non-diabetic obesity adults, Simone et al. showed that prior LVH was significantly associated with an increased risk of subsequent diabetes. In multivariate logistic regression, LVH predicted incident diabetes (*p* < 0.01) after controlling covariates, except that body fat and inflammatory markers were included^23^. However, there are still some confounding factors that need to be addressed in order to establish an association between LVH and DM risk. One reason is that the two studies selected different participants for observation. One study included hypertensive patients, while the other study mainly selected obese individuals, but both studies used diabetes as the outcome. Another reason is that although LVH was defined by LVMI in both studies, their threshold values were different.

Since LVH identified by LVMI has different definitions and the presence or absence of LVH does not demonstrate the extent of left ventricular mass, we considered using LVMI instead of LVH to assess diabetes risk. We use multiple regression analysis to prove an association between high LVMI and risk of diabetes after adjusting for confounders associated with LVH such as BMI, insulin resistance, and hypertension. Besides, some errors from ECG identified left ventricular mass were attributed to biological individual variability and inconsistent QRS voltage due to electrode placement^12^. Consequently, we use Echo to identify left ventricular mass rather than ECG, thus reducing error^24^.

Disagreements between ECG and Echo in identifying LVH could arise due to factors such as gender, ethnicity, age, body composition, and the timing of the examination. ECG-LVH and LVH identified by echo may be distinct due to their representation as electrical and anatomical entities, respectively. Some longitudinal studies showed that abnormal ECG findings can occur earlier than anatomical Echo findings in patients with hypertrophic cardiomyopathy^18,25^. Increasing risk of diabetes is correlated with ECG-LVH or high tertile LVMI, indicating that coexistence has the highest risk. As previously discussed, separating ECG and Echo as distinct entities provides more predictive and practical information for clinicians rather than using each alone.

Recent studies, including ours, have found that LVH may also precede the onset of diabetes. Regarding this finding, some cohort studies have partially elucidated this apparent reverse causal relationship^22^. Our findings are consistent with the CARDA study, which demonstrated a significant positive association between left ventricular mass determined by ECG and the incidence of diabetes, even after adjusting for other risk factors^12^. This unfavorable metabolic phenotype often presents with insulin resistance, increased body fat, and visceral obesity. A 4-year follow-up cohort study investigating the relationship between LVH and the risk of diabetes revealed that LVH precedes incident diabetes and is potentially associated with prior metabolic status, body fat, and inflammation^23^. In cross-sectional and longitudinal studies, patients exhibiting evidence of LVH are sequentially at risk for diabetes which often coexists with metabolic syndrome. Metabolic syndrome involves the clustering of various cardiovascular risk factors and is also recognized as a risk factor for the development of type 2 diabetes^26^. The metabolic syndrome, along with hyperinsulinemia, is commonly linked to obesity and insulin resistance, which are underlying mechanisms for the development of diabetes and hypertension. Elevated insulin levels stimulate the growth of left ventricular mass, regardless of the presence of hypertension or obesity^26^, therefore this suggests that LVH can result from hyperinsulinemia in addition to hypertension or obesity. We hypothesize that a larger left ventricular mass might be a mediator for incident diabetes caused by hyperinsulinemia. In our study, we found that after adjusting for HOMA-IR, higher LVMI remains associated with incident diabetes. This suggests that the association between higher LVMI and risk of diabetes involves the consideration of multiple factors in addition to insulin resistance, including metabolic syndrome, obesity, visceral adiposity and inflammation^26^. In previous research, it was believed that the causal relationship between high left ventricular mass and the risk of diabetes was due to myocardial growth induced by hyperglycemia. Relevant mechanisms included diabetes-induced glycation protein and atherosclerosis^27^. Additionally, the inhibition of the renin-angiotensin system with ACE inhibitors and angiotensin receptor blockers not only regressed LVH, but also delayed the onset of type 2 diabetes. This may be attributed to elimination of insulin resistance and the subsequent improvement of microvascular function^13^.

To the best of our knowledge, this study is the first to comprehensively explore the relationship between ECG or Echo findings and the risk of incident diabetes in a Chinese population. The probability of selection bias was reduced due to the population being from the same community. However, there were some limitations in our study. First, the procedure for identifying LVH electrocardiographically may be influenced by ECG voltage due to individual variability and variability at the site of electrode placement. Second, we were unable to exclude diabetes-related confounding factors such as baseline medications and HbA1c levels, which could have affected our results. Third, although ECG-LVH and high left ventricular mass appear to be distinct entities, it is unclear how the etiology and natural history of diabetes differs between ECG and Echo findings.

In conclusion, we demonstrated a positive association between LVMI tertiles and the risk of incident diabetes, with the highest risk of diabetes observed in the presence of both the uppermost LVMI tertile and ECG-LVH for the Chinese population. Despite their non-invasive nature, easy availability, and convenience for assessment, more supportive evidence is required in the future to prove their application for identifying the risk of diabetes.

## Data Availability

Data access requires a reasonable request, and upon such request, the corresponding author will provide the data.
